# Assessing the influence of changes in land use-land cover on soil properties of degraded sodic lands in Indo-Gangetic plains

**DOI:** 10.1038/s41598-025-30949-8

**Published:** 2025-12-05

**Authors:** Yash Pal Singh, Sanjay Arora, Vinay Kumar Mishra, Ravindra Kumar Gupta

**Affiliations:** 1 ICAR-Central Soil Salinity Research Institute, Regional Research Station, Lucknow, Uttar Pradesh 226002 India; 2 ICAR-Research Complex for North Eastern Hill Region, Umiam, Meghalaya 793103 India; 3 Bohra Institute of Allied Health Sciences, Lucknow, Uttar Pradesh 212208 India

**Keywords:** Land use-land cover, Degraded sodic land, Soil properties, Soil amelioration, Ecology, Environmental sciences

## Abstract

Land use–land cover (LULC) change significantly affects soil fertility and productivity in salt-affected soils. Soil samples across seven LULCs over 20 years (1995–2015) were collected and analysed for ascertaining long-term changes with respect to different land uses. Silvipastoral systems showed the greatest improvement in physico-chemical and biological properties of sodic soil. This system recorded the lowest bulk density (1.44 g cm⁻³), highest porosity (56.34%), and infiltration rate (24.52 mm day⁻¹), along with major reductions in pH, electrical conductivity, and exchangeable sodium percentage (32–54%) indicative of ameliorative potential. Available nutrient N, P and K in surface soil under silvipastoral land use showed an increase of 95.36, 125.60 and 57.0% respectively over the initial values. Build-up of soil organic carbon and microbial biomass carbon content was highest under silvipastoral land use, followed by silviculture and pastoral systems, and lowest in barren land and rice–wheat system. Overall, silvipastoral systems with *Prosopis juliflora* and salt-tolerant grasses (*Leptochloa fusca*, *Trifolium alexandrinum*) proved most effective for improving sodic soil health in the Indo-Gangetic plains.

## Introduction

 Land degradation due to increasing salinity or sodicity is a worldwide problem that needs attention for food security to the increasing population^1^. According to the recent estimates, the total area of salt‑affected soils (SAS) of the world amounts to 1 381 million ha (Mha), or 10.7% of the total global land area befall in many parts of sub-humid, semi -arid, and arid regions of the world^2^. Out of the 329 million hectares geographical area of India, 175 million hectare is degraded and 6.74 million hectares is salt affected land^3^ with excess amounts of soluble (saline) and/or sodic salts, which adversely affect crop/vegetation growth and yield^4,5^. These soils are having high sodium (Na) content and high exchangeable sodium percentage (ESP) which causes dispersion of clay resulting drastic reduction in soil permeability^6^. Due to high dispersion and swelling of clay these soils are unsuitable for crop production^7^. Reclamation and proper management of SAS has been encouraged in developing as well as developed countries^8–11^ and numerous efforts have been made to develop these soils for productive land use systems^12^.

In India, salt affected soils account for about 6.74 million hectares and the future projections indicate an increase in the area of SAS to the extent of about 16 million hectares by 2050 due to inappropriate irrigation and changing climate^3^. Of the total SAS, sodic lands accounts for about 56.3% (3.8 million hectare) in India^13,14^ and as per recent assessment, in the Indo-Gangetic plains, slightly sodic areas increased by 51.8% between 1996 and 2023^15^.

**Fig. 1:**
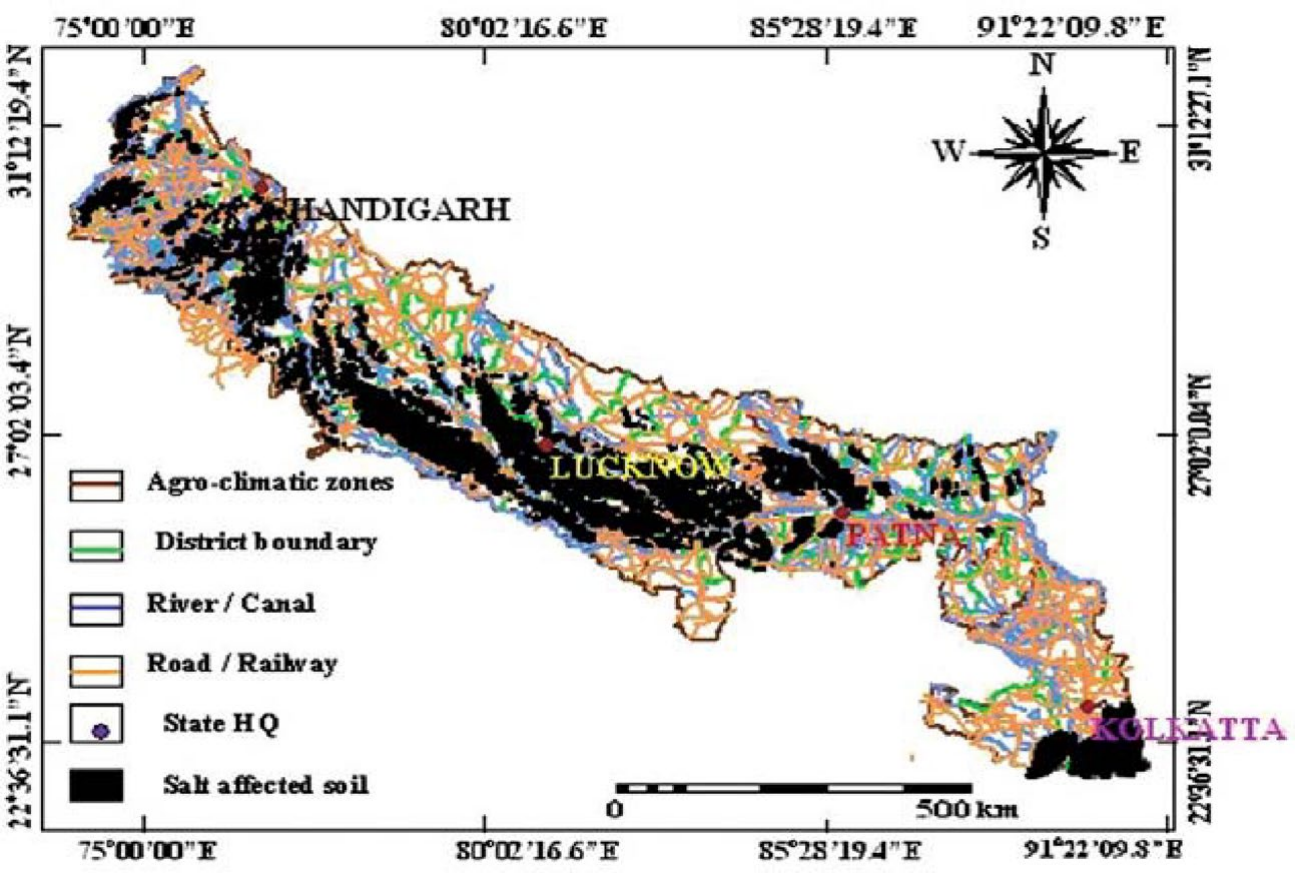
Extent of salt affected soils in Indo-Gangetic plains.

Crop growth and productivity are severely affected due to this problem, resulting in an annual loss of about 4 billion US$. This loss is expected to rise manifold with probable increase of SAS^3,16^. Indo-Gangetic plain zone in India covering five major provinces is highly infested with sodicity problem covering about 2.5 million hectare sodic lands (Fig. 1). Most of the sodic lands in this region are geogenic origin and seepage from canal network subsequently increase salts in the soil profile contributing in augmenting this problem. Salt affected soils in Indo-Gangetic plains is a critical issue for proper land use under the rainfed and irrigated regions^15,17^. During past two decades various efforts have been made to reclaim these sodic soils through chemical amendments using gypsum (CaSO_4_⋅2H_2_O) but it did not gain momentum due to its limited availability in open markets and higher cost^18^. Due to poor soil health conditions, farmers usually allocate share of this land parcel to grow crops, fuel, fodder, and timber for their livelihood security. Among the crops, rice and wheat are the predominant crops cultivated in reclaimed as well as partially reclaimed sodic lands. In certain conditions where irrigation water is not available in abundant quantity, the mono crop of rice in rainy season is practiced and fields are left uncultivated during winter season resulting salts coming up on the surface again. However, biological approaches, such as phytoremediation and plantation of multipurpose tree species and grasses to meet out the fuel, fodder and timber requirement have shown encouraging results in amelioration of sodic soils^19,20^. Changes in land uses lead to a change in the physico-chemical and biological properties of soil. These changes attributed to the anthropogenic activities in the soil surface and sub-surface layers. Soil organic carbon (SOC) is a major constituent to assess the fertility status of sodic soils. The loss of SOC due to different land uses under normal soils is well documented however, in sodic soils it is not scientifically studied because rice-wheat cropping system is predominant in sodic soils^21–24^. On the other hand, the impact of land use changes on soil physico-chemical and biological properties of sodic lands is hardly noticed by the land managers to initiate ameliorative measures^25^. Most of the studies on effect of land use changes on salt affected soils are confined to their ecological restoration however, the present study was planned with the objectives (1) to assess the influence of different land use-land covers on soil physico -chemical and biological properties and (2) find out the changes in nutrient status of sodic soil under different LULCs. For future sustainable land use planning of degraded sodic soils, a long-term study was conducted in Indo-Gangetic plains at the Research Farm of ICAR -Central Soil Salinity Research Institute (CSSRI), Regional Research Station (RRS), Lucknow, India. The hypothesis of the present study was that the different land uses have potential for amelioration of sodic soil through changes in soil properties for which systematic assessment was done.

## Materials and methods

### Study site

**Fig. 2:**
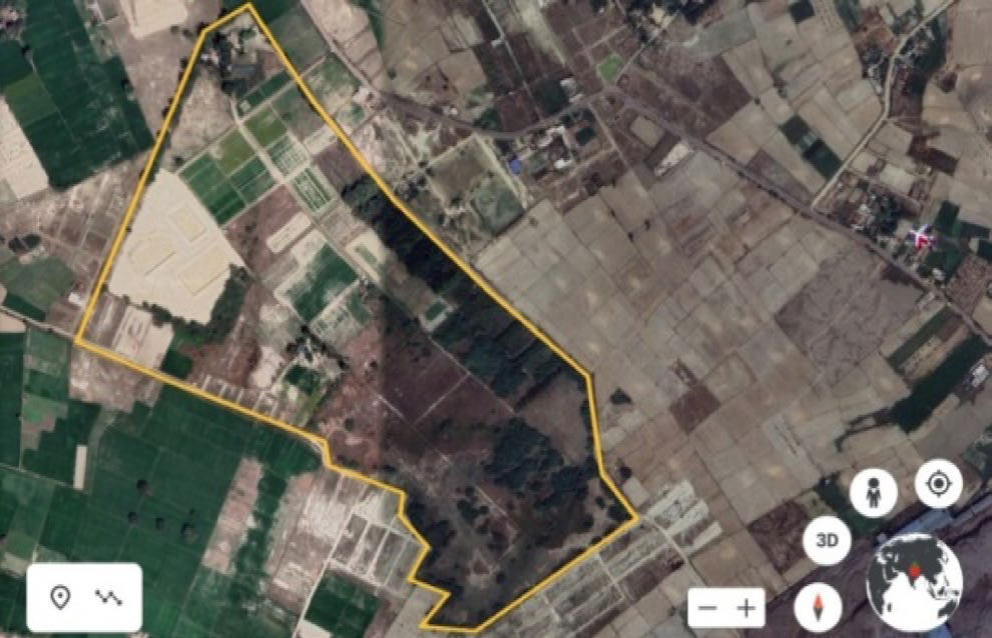
Location map of ICAR-CSSRI, research farm, Shivri, Lucknow, India.

Present study was initiated at the research farm of ICAR—Central Soil Salinity Research Institute, Regional Research Station, Lucknow, U.P., India covering an area of 24 ha sodic land and situated at an elevation of 120 m AMSL representing sodic soils of Central Indo-Gangetic plains. It is located between 26° 47’ 45”− 26° 48’ 13” N and 80° 46’ 7”− 80° 46’ 32” E (Fig. 2). Study site represents a semi-arid sub-tropical climate characterized by hot summer and cold winter. The topography of the research farm resides in the incurvation of moderately sloping plains between 119 and 125 m contours.

**Fig. 3:**
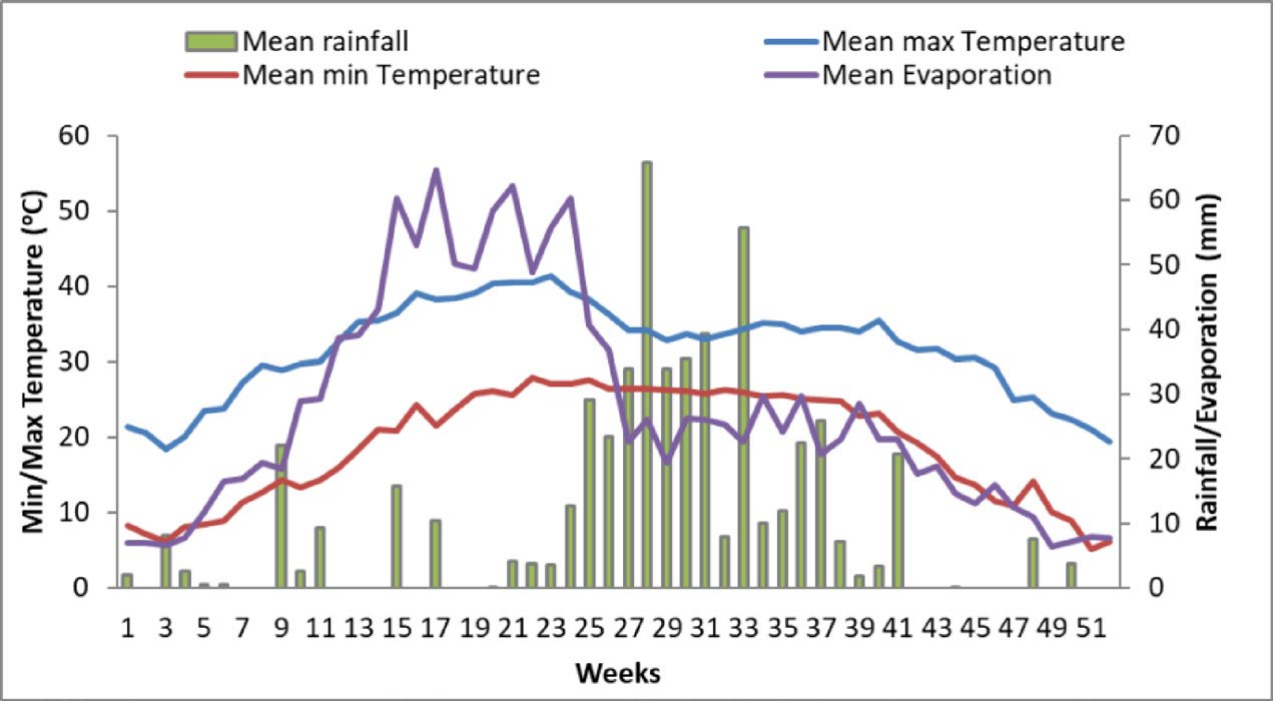
The climatic features (1995–2015) of Shivri Research Farm.

The mean annual rainfall (2000–2020) of the study site is 817 mm that is generally monsoonal occurring between June to October months. The average annual evaporation was 1580 mm. The average maximum and minimum temperatures recorded in the months of May and January were 39 °C and 7.1 °C, respectively (Fig. 3). Thus, temperature regime was hyperthermic and the moisture regime was mainly ustic.

**Fig. 4:**
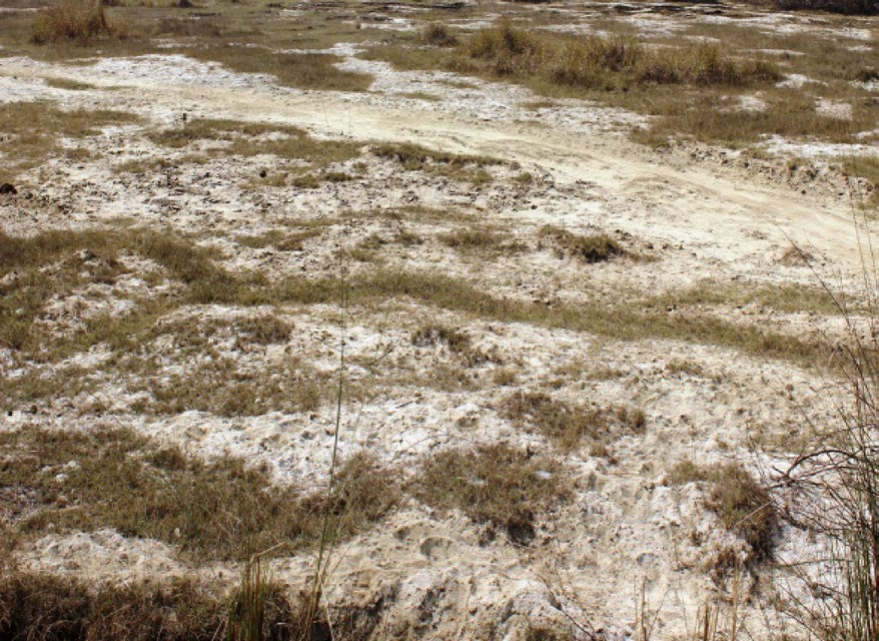
Initial Soil status of experimental site.

The soil was highly sodic (pH_1:2_ 10.5 and ESP 89) fine loamy, mixed, hyperthermic and classified as Aquic Natrustalfs^26^. The site was lying abandoned and has very scanty vegetation with some grass species like *Sporobolus marginatus*, *Cynodon dactylon*, *Sacharum spontanium*, *Aristida obscendens*, *Leptochloa fusca*. *Prosopis juliflora*, *Zizyphus numularia*, *Asparagus racemosus*, *Coculus pendulus* (Fig. 4).

### Experimental design

During 1995 the sodic farmland was divided in to different land parcels. One parcel of the land (1 hectare) was reclaimed for cultivation of rice and wheat using chemical amendments like gypsum (CaSO_4_.2H_2_O) and the remaining 6 ha land was equally divided in to six land parcels for bio-amelioration under different LULCs and put under observation for 20 years. Long-term field experiment consisting of seven different land use land covers viz. (L_1_) (barren grassland), (L_2_) Rice-Wheat, (L_3_) Pastoral (*Panicum maximum*), (L_4_) Silvi-Agriculture (Jatropha + seasonal crops), (L_5_) Silvipastoral (Prosopis + *Laptochloa fusca* followed by *Trifolium alexandrinum*), (L_6_) Silviculture (multipurpose tree species), (L_7_) Horticulture (fruit species) was conducted under Randomized Block Design (Table 1) with five replicate plots.

Five plots of 10 × 10 m size were selected under each LULC for soil sampling. Five sampling points representing whole plot were selected in each plot and samples were collected from three depths (0–15, 15–30 and 30–60 cm) at each sampling point from corresponding LULCs in the month of May. These five samples mixed thoroughly to form a homogenized sample. Accordingly, 15 soil samples collected from each plot and a total 105 (7 × 3 × 5) soil samples were collected from seven LULCs.

**Table 1:** Description of the selected land use-land covers.

Treatments	Land uses	Description about the land uses	
L_1_	Barren grass land (BGL) (Control) (26° 48′ 04″ N, 80° 46′ 21 "E)	The land was covered with natural grasses like *Sporobolus marginatus* and *Cynodon dactylon*. Since, it is not disturbed with any field operation therefore; it is used as reference (Control)	
L_2_	Rice–Wheat (26°48′9.3" N, 80°46′ 22.0" E)	The land parcel selected for this land use was reclaimed with gypsum and brought under cultivation of Rice–wheat system (salt tolerant varieties) since year 1995 (> 20 years) with use of inorganic fertilizers (DAP, Urea, MOP and Zinc sulphate) and irrigated	
L_3_	Pastoral (*Panicum maximum)* (26°48′1.6" N, 80° 46′16.0″ E)	This perennial fodder species (*P. maximum*) was planted in a land parcel of highly sodic soils without use of any chemical amendments under rainfed condition to monitor the effect of grass species on amelioration of sodic soils for > 20 years. This fodder species was harvested regularly and used as fodder for animals	
L_4_	Silvi-Agriculture (Jatropha + seasonal agricultural crops) (26°47′1.3" N, 80°46′31.5" E)	This land was covered under Jatropha plantation and various inter crops were grown in between Jatropha rows under sodic soil condition. The seasonal crops include, Ocimum-Matricaria during rabi season and maize-linseed during kharif seasons	
L_5_	Silvipastoral (*Prosopis juliflora* + grasses) (26° 47′58"N, 80° 46′24" E)	The selected land was covered under *P. juliflora* with intercropping of grass species like *L. fusca* (L.) followed by *T. alexandrinum* without any chemical amendments	
L_6_	Silviculture (Multipurpose tree species) (26° 48′ 04″ N, 80° 46′ 21 "E)	This land use parcel was covered under multipurpose tree species like *P. juliflora, A. nilotica, C. equsatifolia, P. pinnata, P. dulce, E. tereticornis, A.nilotica, A.indica, T.arjuna, and P.alba* and no field operation is being done for > 20 years. The surface is covered under natural vegetation	
L_7_	Horticulture (fruits) (26°48′1.6" N, 80° 46′16.0″ E)	This land use parcel was covered under 8 fruit tree species like *P. granatum, S. guajava, C. karanda, M. indica, T. indica, S. cuminai, P. emblica, and Z. mauritiana,* and no inorganic fertilizer is used since year 1995. During initial stage of plant growth limited tillage operations and irrigation were done	

The water samples from different irrigation sources viz. tube wells and hand pumps available at the farm were collected and analysed for quality appraisal. Three water samples were collected from tube wells, two of them from a depth of 55 m and one from 110 m. Two samples were collected from the hand pumps of 20 m depth installed at the farm. The irrigation water used for different LULCs was having low electrolyte concentration (0.56–0.72 dS m^− 1^) and pH (7.50–8.29) which were quite safe for irrigation in all types of crops. Among the cations, Na^+^ dominates over Ca^++^ and Mg^++^ followed by K^+^ whereas, HCO_3_^−^ plus CO_3_^−^ anions dominates over Ca^++^, and SO_4_^2−^ are absent. The residual alkalinity in these waters ranged from 1.3 to 1.7 me l^− 1^ and SAR from 2.9 to 3.3 me l^− 1^ (Table 2). Prolonged irrigation with such waters may cause slow alkalization of soils.

**Table 2:** Water quality of study site.

Sample no.	Source of water	Depth of water (m)	pH	EC (dS m^− 1^)	CO_3_^−^	HCO_3_^−^	Cl^−^	SO_4_^2−^	Ca^++^ Mg^++^	Na^+^	K^+^	RSC	SAR
					me l^− 1^								
W_1_	T_1_	55	7.56	0.68	1.2	4.0	2.1	0.0	3.7	4.1	0.12	1.5	3.0
W_2_	T_2_	110	8.08	0.57	1.2	3.2	1.5	0.0	3.1	3.7	0.12	1.3	2.9
W_3_	H_1_	20	7.50	0.56	1.2	3.1	2.6	0.0	3.2	3.8	0.16	1.4	2.9
W_4_	H_2_	20	8.29	0.72	1.3	4.0	2.3	0.0	3.6	4.4	0.11	1.7	3.3

T_1_-Tubewell 1, T_2_-Tubewell 2, H_1_- Hand pump 1, H_2_-Handpump 2; RSC: Residual sodium carbonate; SAR: Sodium adsorption ratio.

### Initial soil properties of the study site

#### Soil mineralogy of the study site

Semi quantitative estimates of sand, silt and clay minerals in sodic soils of experimental site were conducted before initiating the LULC studies. From the analysis it was found that the Quartz (0.426 nm) mineral dominates in sand followed by plagioclase feldspars (0.321 to 0.328 nm), mica (1.0 nm), chlorite (1.4 nm), orthoclase feldspars (0.312 to 0.323 nm), amphibole (0.845 nm) and mixed layer minerals (1.21 nm). Calcite (0.303 nm) was absent in the sand fraction of A horizon but was present in minute quantities in B horizon. The silt fraction mineralogy was similar to that of the sand fraction with an exception that the mica increased considerably in silt. General order of mineral preponderance was mica, plagioclase, quartz, orthoclase, chlorite, kaolinite, mixed layer, calcite and amphibole. According to semi-quantitative XRD for clay minerals, the as cendency of illite, followed by chlorite, kaolinite, mixed layer and smectite minerals were estimated^26^ (Table 3).

**Table 3:** Semiquantitative estimates of clay minerals (%) in sodic soils of Shivri experimental farm.

Sample no.	Smectite	Vermiculite	Chlorite	Mixed layer	Illite	Kaolinite
1 (A-Horizon)	7	–	12	7	65	9
2 (B-Horizon)	4	–	15	11	56	14

#### Methodology to assess soil properties

To monitor the initial soil properties of the study site, eight soil profiles were dugout up to a depth of 120 cm at the farm in 1995, collected soil samples from each horizon and analysed for soil physico-chemical and biological properties. Soil bulk density (BD) was computed from intact soil samples by weighing a known volume of a 5.3 cm internal diameter core sampler^27^. Water holding capacity (WHC) and soil porosity (SP) were determined following the procedure recommended by keens box^28^. Double concentric infiltrometer cylinder with 60 cm outer and 30 cm inner diameters was used to measure Infiltration rate (IR)^29,30^. The observation of first water fall was recorded after 5 min and the subsequent observations were taken after 10, 20, 30, 40, 50, 60, 90 and 120 min of ponding water. The homogenized samples were dried and passed through a 2.0 mm sieve and used for analyzing soil properties. Soil pH and electrical conductivity (EC) were determined in 1:2 soil: water ratio. Digital pH and conductivity meters were used to measure soil pH and EC using 1:2 soil water suspension^31^. Exchangeable sodium percentage (ESP) was computed following the method^32^. Organic carbon (OC) content was analyzed through rapid titration method described by^33^. Available nitrogen was assessed by soil distillation with KMnO_4_ and NaOH^34^, available P and K by the Olsen’s sodium bicarbonate extraction^35^ and sodium acetate extraction methods, respectively. The Flame photometer was used to measure Na^+^ and K^+^ concentrations in soil saturation extract and the concentration of Ca^2+^ and Mg^2+^ in soil extract was estimated by the Versenate titration method^36^. Titration method with 0.01 N H_2_SO_4_ using phenolphthalein and methyl orange indicators was used to determine carbonate (CO_3_^-^) and bi-carbonate (HCO_3_^-^ ) contents in soil extract. Soil MBC was determined using fumigation extraction method^37^ and MBN by CHCl_3_ fumigation extraction technique^38^. The soil MBP was measured using chloroform fumigation extraction method^39^ and the triphenyl tetrazolium chloride (TTC) method^40^ was used to determine soil dehydrogenase activity (DHA). The average initial physico-chemical and biological properties of the study site are given in Table 4.

**Table 4:** Initial properties (year 1995) of the study site (*N* = 8).

Soil parameters	Soil depth (cm)				
	0–15	15–30	30–60	60–90	90–120
Sand (%)	62.40 ± 1.45	49.60 ± 2.10	48.30 ± 0.80	57.00 ± 1.20	52.30 ± 0.90
Silt (%)	19.80 ± 0.61	25.40 ± 0.75	20.90 ± 0.70	23.40 ± 0.80	25.50 ± 1.20
Clay (%)	17.80 ± 0.60	25.00 ± 0.80	30.80 ± 1.10	19.60 ± 0.90	22.20 ± 1.00
Bulk density (g cm^− 3^)	1.60 ± 0.01	1.57 ± 0.02	1.53 ± 0.01	1.50 ± 0.04	1.49 ± 0.02
Porosity (%)	42.40 ± 0.60	44.20 ± 0.50	44.40 ± 0.30	42.20 ± 1.00	38.50 ± 0.60
WHC (g kg^− 1^)	3.20 ± 0.02	3.60 ± 0.03	3.80 ± 0.02	3.80 ± 0.03	3.30 ± 0.01
Infiltration rate (mm day^− 1^)	4.00 ± 0.02	–	–	–	–
pH_1;2_	10.50 ± 0.10	10.40 ± 0.20	10.40 ± 0.10	10.00 ± 0.20	9.60 ± 0.20
EC_1:2_ (dS m^− 1^)	2.42 ± 0.10	1.43 ± 0.12	0.86 ± 0.03	0.64 ± 0.04	0.45 ± 0.01
ESP	89 ± 2.50	91 ± 4.00	85 ± 2.60	80 ± 2.50	60 ± 3.20
SOC (g kg^− 1^)	0.80 ± 0.02	0.80 ± 0.03	0.60 ± 0.02	0.60 ± 0.02	0.60 ± 0.02
Ca^2+^ + Mg^2+^ (me l^− 1^)	2.10 ± 0.21	2.60 ± 0.12	1.60 ± 0.06	1.60 ± 0.02	2.10 ± 0.01
Na^+^ (me l^− 1^)	11.70 ± 0.07	24.90 ± 0.11	25.60 ± 0.06	19.30 ± 0.07	4.10 ± 0.02
K^+^ (me l^− 1^)	0.05 ± 0.01	0.06 ± 0.001	0.01 ± 0.001	0.01 ± 0.001	0.04 ± 0.001
CO_3_^−^ (me l^− 1^)	0.00±	6.00 ± 0.04	2.50 ± 0.02	4.00 ± 0.03	1.00 ± 0.002
HCO_3_^−^ (me l^− 1^)	12.50 ± 0.15	15.50 ± 0.34	22.00 ± 0.43	11.00 ± 0.32	3.00 ± 0.01
Cl (me l^− 1^)	2.00 ± 0.03	3.50 ± 0.02	7.00 ± 0.02	3.00 ± 0.01	3.00 ± 0.01
SO_4_ (me l^− 1^)	0.00 ± 0.001	0.60 ± 0.002	0.50 ± 0.001	3.30 ± 0.002	11.00 ± 0.06
CaCO_3_ (g kg^− 1^)	14.10 ± 2.10	12.60 ± 1.20	23.20 ± 2.00	23.20 ± 4.00	37.70 ± 0.90
Alkaline KMnO_4_–N (kg ha^− 1^)	94.00 ± 1.20	62.72 ± 1.00	54.60 ± 1.50	45.10 ± 1.43	40.60 ± 1.20
Olsen’s P (kg ha^− 1^)	25.00 ± 0.40	21.60 ± 0.50	18.50 ± 0.40	17.00 ± 0.32	16.10 ± 0.20
Available K (kg ha^− 1^)	388.80 ± 22.5	384.00 ± 21.20	321.40 ± 12.40	238.60 ± 16.40	169 ± 11.20
Bacterial count (cfu g^− 1^)	6.3 × 10^4^ ± 0.33				
Fungal count (cfu g^− 1^)	2.14 × 10^3^ ± 0.12				
MBC (µg g^− 1^)	24.34 ± 1.23				
MBN (µg g^− 1^)	2.32 ± 0.22				
MBP (µg g^− 1^)	4.45 ± 0.14				
Dehydrogenase activity (TPFg^− 1^h^− 1^)	1.38 ± 0.06				

*pH*_*1:2*_ *and EC*_*1:2*_ refers to soil: water suspension ratio of 1:2, *ESP* exchangeable sodium percentage, *BD* bulk density, *IR* infiltration rate; *WHC*, water-holding capacity, *SOC* soil organic carbon, *MBC* microbial biomass carbon, *MBN* microbial biomass nitrogen, *MBP* microbial biomass phosphorus.

### Data analysis

The statistical analysis of experimental data was carried out using SPSS 18.0 to test the effects of LULCs on soil physio-chemical and biological properties using the two-way ANOVA. The Pearson’s multiple correlation analysis was carried out to find out the relationship between soil parameters under different LULCs. Least significance difference (LSD) test at *p* < 0.05 was used to analyze the comparison between the treatment means. All data are an average of five replicates ± SE of the composite soil samples. The principal component analysis (PCA) which limits the variables and extracts smaller number of independent factors (principal components) for deducing the association among observed variables was performed by varimax rotation with Kaiser Normalization. Varimax rotation was employed because orthogonal rotation minimizes the number of variables with a high loading on each component and therefore facilitates the interpretation of PCA results. The number of significant principal components were selected in view of the Kaiser criterion with eigenvalue greater than 1^41^. The analysis will help in observing the trend of changes in soil properties under different LULCs uncovering the relationships between observations and variables and among the variables.

## Results

### Effect of different land use-land covers on soil physical properties

The soil physical properties including sand, silt, clay content, BD, SP, WHC, and IR were significantly (*p* ≤ 0.05) influenced by the different LULCs (Table 5). The sand and silt contents ranges from 60.10% to 68.50% and 19.50% to 24.40%. The soil under rice-wheat land use was considerably higher in sand content and lowest in clay content. It was followed by the silvi -agriculture, pastoral and horticulture LULCs. The reduction in clay content under different land uses ranges 2.8% to 36.68%. The highest reduction (36.68%) in this soil textural fraction was recorded under continuous use of land parcel under rice-wheat land use. This was followed by silvi-agriculture (20.78%) and horticultural (20.22%) LULCs. The silt content was higher in silvi-pastoral followed by silviculture and pastoral land uses. The silt content after 20 years of silvi -pastoral and silvicultural land uses increased to the level of 23.23%, and 8.1% over the initial values and 20.60% and 5.8% over traditional rice -wheat land use, respectively. The results indicated that the mean BD (g cm^-3^) of sodic soils was significantly (*p* ≤ 0.05) influenced by the different LULCs and over the period of study. Among the land uses studied, the BD in agroforestry LULCs was significantly (*p* = 0.005) different from BGL and rice –wheat land uses. The highest reduction from initial value of bulk density 1.60 g cm^-3^ in surface soil was recorded in silvi-pastoral land use (1.44 ± 0.002 g cm^-3^) followed by silviculture, horticulture, pastoral, and silvi-agricultural LULCs; whereas, the lowest (1.56 ± 0.002 g cm^-3^) in rice-wheat land use. From the data given in Table 9 it was revealed that there was a negative correlation with soil organic carbon and positively correlated with clay content. The soil porosity ranged from 42.05% to 56.34% across all LULCs compared to initial value of 42.4%. The highest (56.34 ± 1.13%) porosity was recorded under silvi-pastoral followed by silviculture (52.20 ± 1.36%), pastoral (45.44 ± 0.85%), and horticulture (44.22 ± 0.65%) LULC; while the lowest (43.63 ± 1.12%) under rice–wheat (Table 5). Soil infiltration rate (IR) also affected significantly (*p* ≤ 0.05) under different LULCs. In our study infiltration rate in different land uses ranged from 11.80 mm to 24.52 mm day^-1^ after 20 years compared to initial value of 4.0 mm day^-1^. Maximum improvement in this parameter was documented under silvi-pastoral land use followed by silviculture, horticulture and pastoral and the minimum under BGL and rice-wheat land uses. The IR of sodic soils under agroforestry LULCs increased to more than 20% over the traditional rice-wheat land use (Table 5).

**Table 5:** Effect of land use-land covers on soil physical properties (*n* = 10).

Land use-land covers	Sand (%)	Silt (%)	Clay (%)	Bulk density (g cm^− 3^)	Porosity (%)	Water holding capacity (mm m^− 1^)	Infiltration rate (mm day^− 1^)
Barren grass land (Control)	68.50a	20.23b	11.27c	1.56a	43.63c	34.22b	16.23a
Rice-Wheat	64.57a	21.00b	14.43b	1.46a	45.44c	38.72a	19.60a
Pastoral	65.80a	20.10b	14.10b	1.53a	45.10c	43.20a	21.40a
Silvi-Agriculture	60.10a	24.40a	15.50b	1.44a	56.34a	46.20a	24.52a
Silvipastoral	63.50a	21.40b	15.10b	1.45a	52.20b	41.65a	24.17a
Silviculture	65.40a	20.40b	14.20b	1.46a	44.22c	40.12a	21.30a
Horticulture	62.40a	19.80b	17.80a	1.64a	40.70d	23.40c	2.10b
Initial	68.50a	20.23b	11.27c	1.56a	43.63c	34.22b	16.23a
SE±	1.43	0.46	0.51	0.03	0.57	1.16	1.36
LSD (0.05)	4.46	1.52	1.63	0.08	1.68	3.63	4.32

*LSD* least significant difference, *BD* bulk density, *IR* infiltration rate, *WHC* water holding capacity, *SE* standard error.

Statistical significance letters (a, b, c, d) assigned using LSD (0.05).

### Effect of different land use-land covers on soil chemical properties

**Fig. 5:**
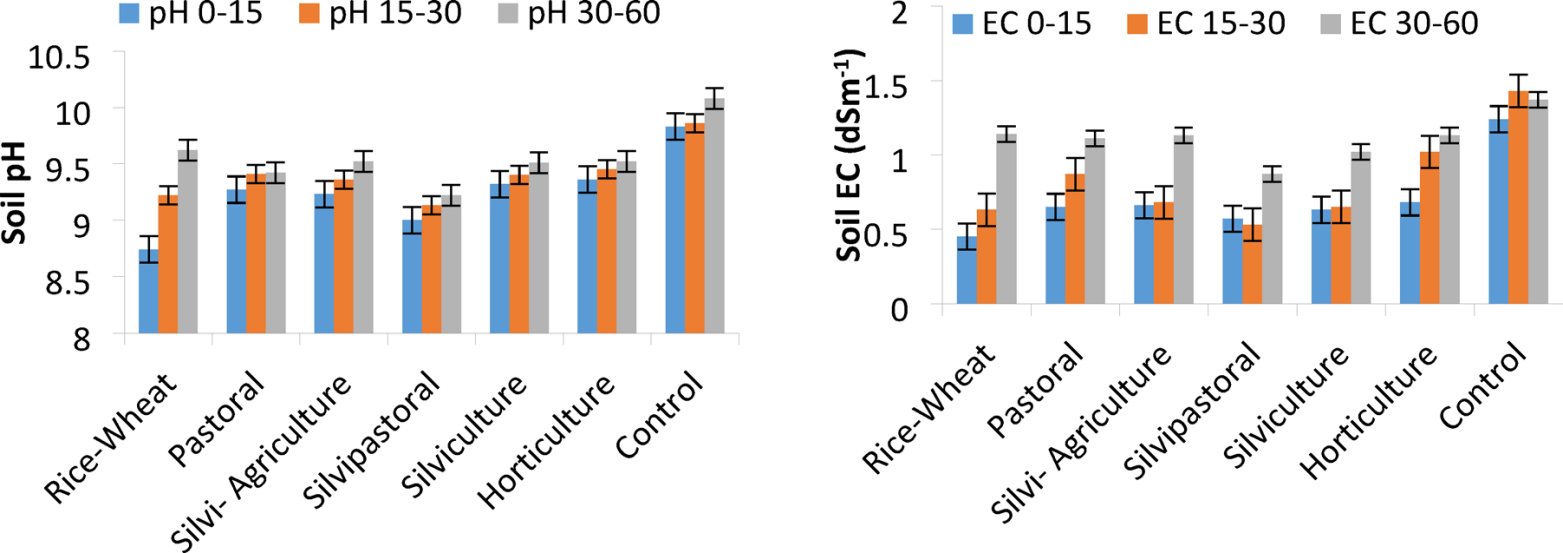

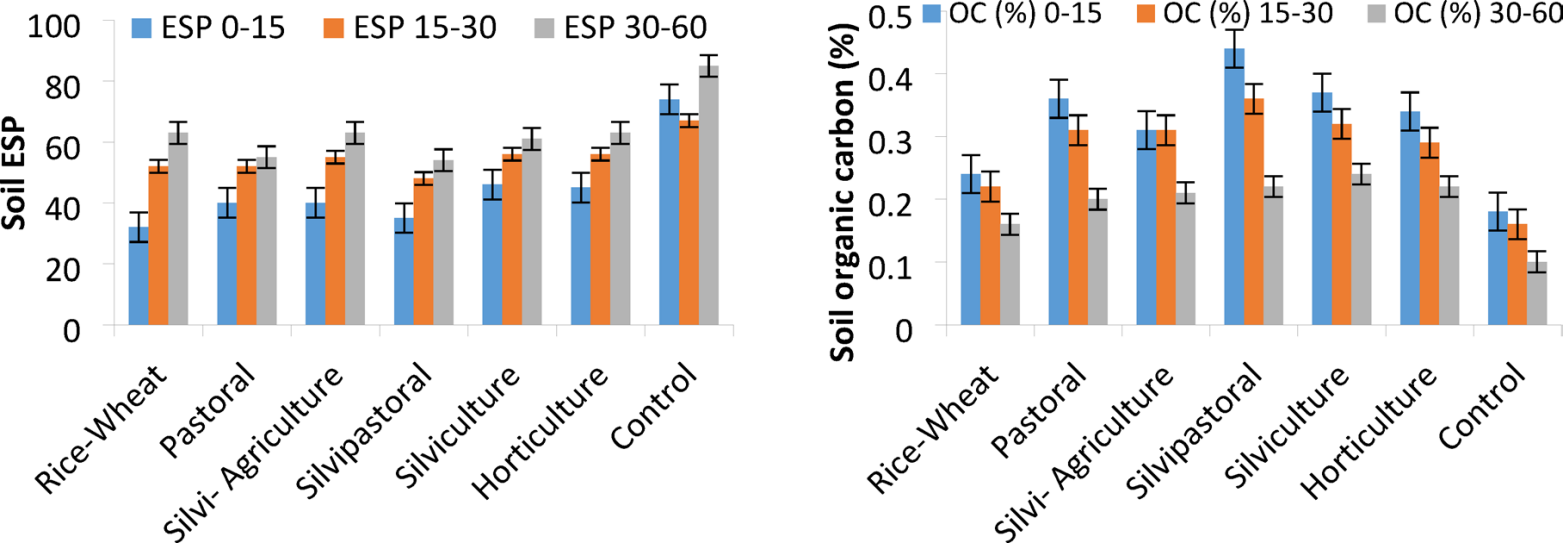
Effect of land use-land covers on soil pH, EC, ESP and SOC (*n* = 10) Bars showing SE.

Soil pH_1:2_, electrical conductivity (EC_1:2_), ESP, and SOC were significantly (*p* ≤ 0.05) affected by different LULCs. The pH_1:2_ of all the land parcels used for different LULCs for 20 years was still within the sodicity level (8.74–10.08) although reduced from the initial value of 10.50 in surface soil. The pH_1:2_ of surface soil (0–15 cm) of land parcel put under continuous cultivation of rice-wheat for 20 years was significantly lower than the land parcels covered under agroforestry land use and BGL. However, under sub-surface soils (15–30 and 30–60 cm) highest reduction in soil pH_1:2_ was recorded under silvipastoral land use. Soil EC_1:2_ of surface soil (0–15 cm) under different land uses ranges from 0.45 to 1.43 dSm^-1^ and it increased with the increasing soil depth. Maximum reduction in soil EC_1:2_ in surface (76%) soil was recorded in rice-wheat land use and in sub-surface (63%) under silvipastoral LULC followed by silviculture and pastoral land uses. The soil ESP increased with increasing soil depth but there was significant reduction in ESP irrespective of soil depth in all the land uses over the BGL. The ESP of surface soil before start of experiment was 89. Among the land uses, silvipastoral LULC showed 32%, 48%, and 54% reduction in ESP at 0–15, 15–30, and 30–60 cm soil depths, respectively, which was significantly lower than rest of the land uses (Fig. 5). The SOC content in all three soil depths (0–15, 15–30 and 30–60 cm) varied significantly under different LULCs. It decreased with increasing soil depth across the land uses. The highest improvement in SOC content at all three soil depths was recorded under silvipastoral (0.44, 0.36. 0.28%) followed by silvicultural (0.37, 0.31, 0.24%) LULCs and the lowest in BGL and rice-wheat land uses. Among the LULCs, the accumulation of SOC under agroforestry land uses was more pronounced up to 30 cm depth and beyond that, the difference between the LULCs was not statistically significant.

### Effect of different land use-land covers on soil microbial properties

**Fig. 6:**
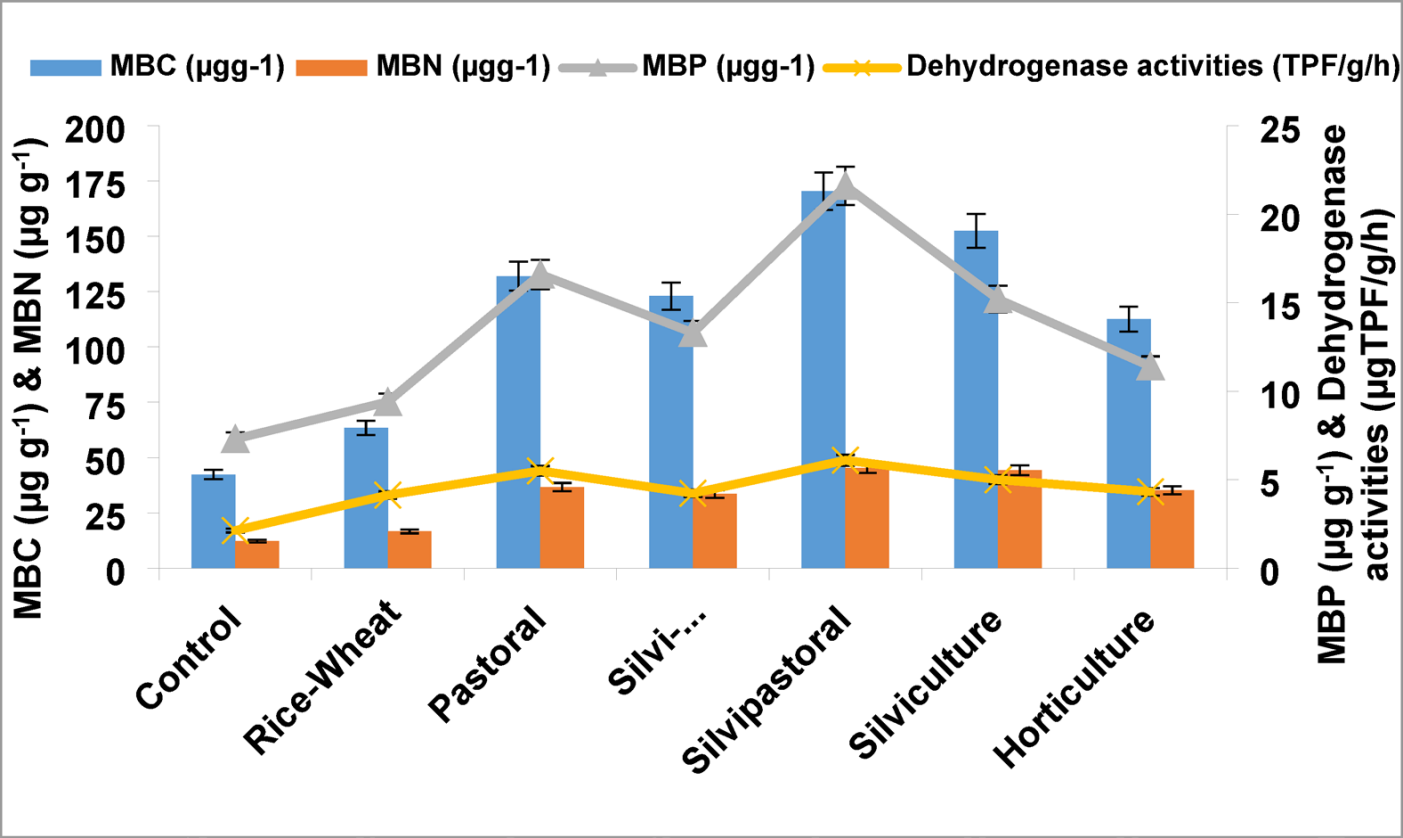
Effect of different land use-land covers on soil microbial properties.

Different LULCs significantly influenced the microbial properties of sodic soil. The mean MBC, MBN and MBP varied from 42.3 to 170.4, 12.2 to 45.3, and 7.3 to 21.6 µg g^− 1^, respectively in 0–15 cm soil layer across the land uses (Fig. 6). The highest concentrations of MBC, MBN and MBP were recorded in silvipastoral followed by silviculture and pastoral land uses. The MBC in agroforestry-based LULCs exhibited a significant difference (*p* ≤ 0.05) over the BGL and rice-wheat land uses. The soil of silvipastoral land use had the highest mean value (170.4 µg g^− 1^) of MBC, followed by silviculture (152.5 µg g^− 1^) and pastoral (132.0 µg g^− 1^) and the lowest (42.3 µg g^− 1^) in BGL and rice-wheat (63.4 µg g^− 1^) compared to initial value of 24.34 µg g^− 1^. In our study, DHA varied significantly among the land uses. The highest values of DHA (6.1 µg TPF g^− 1^ h^− 1^) was observed in silvipastoral followed by pastoral (5.5 µg TPF g^− 1^ h^− 1^) and silviculture land uses in comparison to DHA of 1.38 TPF g^− 1^ h^− 1^ in the initial composite soil sample. Minimum DHA content was recorded in BGL (2.13 TPF g^− 1^ h^− 1^) and rice-wheat (4.13 µg TPF g^− 1^ h^− 1^) land uses.

### Effect of different land use land covers on soil fertility

Available nitrogen content in soil under different LULCs ranged from 94.56 to 183.64, 92.20 to 143.20, and 65.40 to 83.40 kg ha^-1^ at 0–15, 15–30, and 30–50 cm soil depths, respectively. It was found to be higher in all the LULC compared to the intial value of 94 kg ha^-1^. The highest mean available nitrogen contents irrespective of soil depths were recorded under silvipastoral followed by silviculture LULCs, which was significantly higher over the BGL, and rice-wheat land uses (Table 6). The available N content in surface soil was found to be 95.36% higher in silvipastoral land use compared to initial soil content. Available phosphorus and available potassium in the study site were also significantly affected with different LULCs over the initial content of 25 and 388.8 kg ha^-1^, respectively. Available P and K in agroforestry based LULCs were significantly higher over BGL and rice-wheat land uses. The data given in Table 6 indicated that among the LULCs, the highest amounts of available N and P irrespective of soil depths were recorded in silvipastoral LULC. However, the level of available P in 0–15 (38.30 kg ha^-1^) and 15–30 cm (31.50 kg ha^-1^) soil depths in rice-wheat land use was at par with pastoral because of continuous application of phosphatic fertilizers in rice-wheat land use. It was computed that in surface soil, available P content was build up to the tune of 6, 53.2, 45.6, 66.0, 125.60, 72.8 and 29.8 per cent over the initial content in control, rice-wheat, pastoral, silvi-agriculture, silvipastoral, silviculture and horticulture land use, respectively. Similarly, available K content in surface soil was 57% higher than the initial value in silvipastoral land use followed by 38.9% in silviculture, 34.36% in silvi-agriculture, 30.97% in pastoral, 24.10% in horticulture, 11.24% in rice-wheat and 0.93% in BGL land use.

**Table 6:** Effect of land use-land covers on soil fertility (*n* = 10).

Land use-land covers	Available *N* (kg ha^− 1^)			Available *P* (kg ha^− 1^)			Available K (kg ha^− 1^)		
	0–15	15–30	30–60	0–15	15–30	30–60	0–15	15–30	30–60
Control	94.56 ± 4.23 f	86.20 ± 4.32 e	53.40 ± 3.37 e	26.50 ± 2.54 e	19.50 ± 2.14 e	16.40 ± 1.12 e	392.40 ± 13.12 f	265.60 ± 6.23 f	240.30 ± 8.33 f
Rice-Wheat	123.30 ± 6.13 e	92.44 ± 4.23 de	54.22 ± 3.12 e	38.30 ± 2.12 d	31.50 ± 1.13 c	19.70 ± 1.13 d	432.50 ± 23.22 e	312.30 ± 11.34 e	269.50 ± 6.12 e
Pastoral	160.97 ± 4.34 d	112.40 ± 7.44 cd	65.35 ± 3.45 d	36.40 ± 2.22 d	29.70 ± 2.12 cd	23.40 ± 1.63 b	509.20 ± 12.64 d	403.60 ± 9.63 d	296.40 ± 7.23 d
Silvi- agriculture	164.23 ± 5.12 cd	123.40 ± 6.12 c	68.20 ± 2.34 cd	41.50 ± 1.43 c	31.60 ± 1.45 bc	21.70 ± 1.47 c	522.40 ± 14.33 cd	412.30 ± 7.54 cd	342.50 ± 6.36 c
Silvipastoral	183.64 ± 7.14 a	143.20 ± 5.22 a	83.40 ± 3.34 a	56.40 ± 3.12 a	33.60 ± 2.23 a	24.60 ± 1.32 a	610.40 ± 12.25 a	540.20 ± 8.67 a	365.60 ± 7.12 a
Silviculture	172.40 ± 8.12 b	124.20 ± 4.33 bc	76.30 ± 4.12 b	43.20 ± 2.23 bc	32.70 ± 1.45 ab	23.50 ± 2.12 ab	540.30 ± 16.34 bc	453.60 ± 11.22 b	312.20 ± 8.44 bc
Horticulture	168.30 ± 5.34 bc	116.10 ± 3.63 cd	66.30 ± 4.37 d	32.45 ± 1.67 de	23.40 ± 2.12 d	22.50 ± 1.43 bc	482.50 ± 12.23 de	376.40 ± 8.23 de	287.60 ± 6.12 de
SE±	2.12	2.34	1.13	1.47	0.68	1.12	4.03	2.74	2.36
LSD _(*p* ≤ 0.05)_	6.13	7.32	3.35	4.63	2.13	3.31	12.43	8.46	7.23

### Effect of different land use land covers on ionic content in soil

There were significant (*p* ≤ 0.05) variations in ionic contents under different LULCs (Table 7). The level of Ca + Mg in 0–15, 15–30 and 30–60 cm soil depths was highest under L_5_ (Silvipastoral) followed by L_6_ (silviculture) LULCs whereas, the lowest in BGL and rice-wheat land uses. The K and Na contents under different LULCs ranges from 0.11 to 0.43 and 2.15 to 8.55 meq l^-1^, respectively. The maximum K content in 0–15, 15–30 and 30–60 cm soil depths was recorded under treatment L_5_ followed by L_2_. The Na content at all the soil depths was lowest in L_5_ followed by L_6_ LULCs whereas, the highest in BGL and rice-wheat land uses. Similar trend was observed in Cl contents. After 20 years of different LULCs, the levels of CO_3_ and HCO_3_ contents varied from 1.33 to 6.33 and 1.00 to 16.30 meq l^-1^, respectively. The average maximum CO_3_ content was recorded under BGL and rice-wheat land uses whereas, the minimum under L_5_ and L_4_ LULCs. However, the HCO_3_ content in surface soil (0–15 cm) was lowest under L_3_ followed by L_2_ and L_1_ whereas, under 15–30 and 30–60 cm soil depths it was lowest under L_3_ and L_2_ land uses (Table 7).

**Table 7:** Effect of different land use-land covers on ionic content.

Ionic content (meq l^− 1^)	Soil Depth (cm)	L_1_	L_2_	Land uses							
				L_3_	L_4_	L_5_	L_6_	L_7_	Mean	SE±	LSD _(0.05)_
Ca + Mg	0–15	4.83d	5.66c	7.83b	7.50b	11.23a	8.33b	6.17c	7.36	0.800	0.63
	15–30	7.00d	7.66c	7.50c	6.83d	10.30a	8.50b	8.33b	8.02	0.447	0.28
	30–60	6.83b	5.00b	4.16b	6.17b	8.65a	8.00a	5.67b	6.35	0.605	1.13
K	0–15	0.23c	0.42a	0.36b	0.33b	0.43a	0.31b	0.28b	0.34	0.027	0.04
	15–30	0.18c	0.27a	0.17c	0.21b	0.27a	0.16c	0.16c	0.20	0.018	0.02
	30–60	0.14a	0.14a	0.11c	0.13b	0.13b	0.11c	0.11c	0.12	0.005	0.01
Na	0–15	8.55a	4.50b	3.32b	4.20b	2.15b	3.23b	4.21b	4.31	0.769	1.13
	15–30	7.43a	7.80a	5.23b	7.20a	4.15c	4.21c	7.16a	6.17	0.599	0.46
	30–60	8.36b	10.50a	7.16c	8.16b	5.12d	4.16e	8.13b	7.37	0.808	0.63
Cl	0–15	8.17a	6.67b	8.16a	6.33b	4.17c	5.42b	8.00a	6.70	0.581	1.06
	15–30	6.83b	5.50b	6.00b	8.83a	3.43c	7.33b	8.83a	6.68	0.726	1.23
	30–60	6.83b	5.33b	6.17b	9.17a	3.00c	5.50b	8.33a	6.33	0.773	1.21
CO_3_	0–15	6.33a	5.33b	5.33b	2.67d	2.23e	4.00c	4.00c	4.27	0.564	0.41
	15–30	5.33a	3.33b	1.67d	1.67d	1.33e	2.33c	3.23b	2.70	0.528	0.23
	30–60	3.33b	4.00a	4.00a	1.33d	1.33d	3.33b	2.67c	2.86	0.430	0.36
HCO_3_	0–15	16.30a	3.83c	3.50c	4.67c	4.23c	4.83c	8.66b	6.57	1.746	2.12
	15–30	7.00a	3.67c	1.83c	4.83b	3.13c	2.17c	5.17b	3.97	0.690	1.06
	30–60	12.00a	0.50e	4.00c	1.00e	4.23c	2.01d	5.50b	4.18	1.474	0.76

Statistical significance letters (a, b, c, d) assigned using LSD (0.05).

### Principal component analysis

**Fig. 7:**
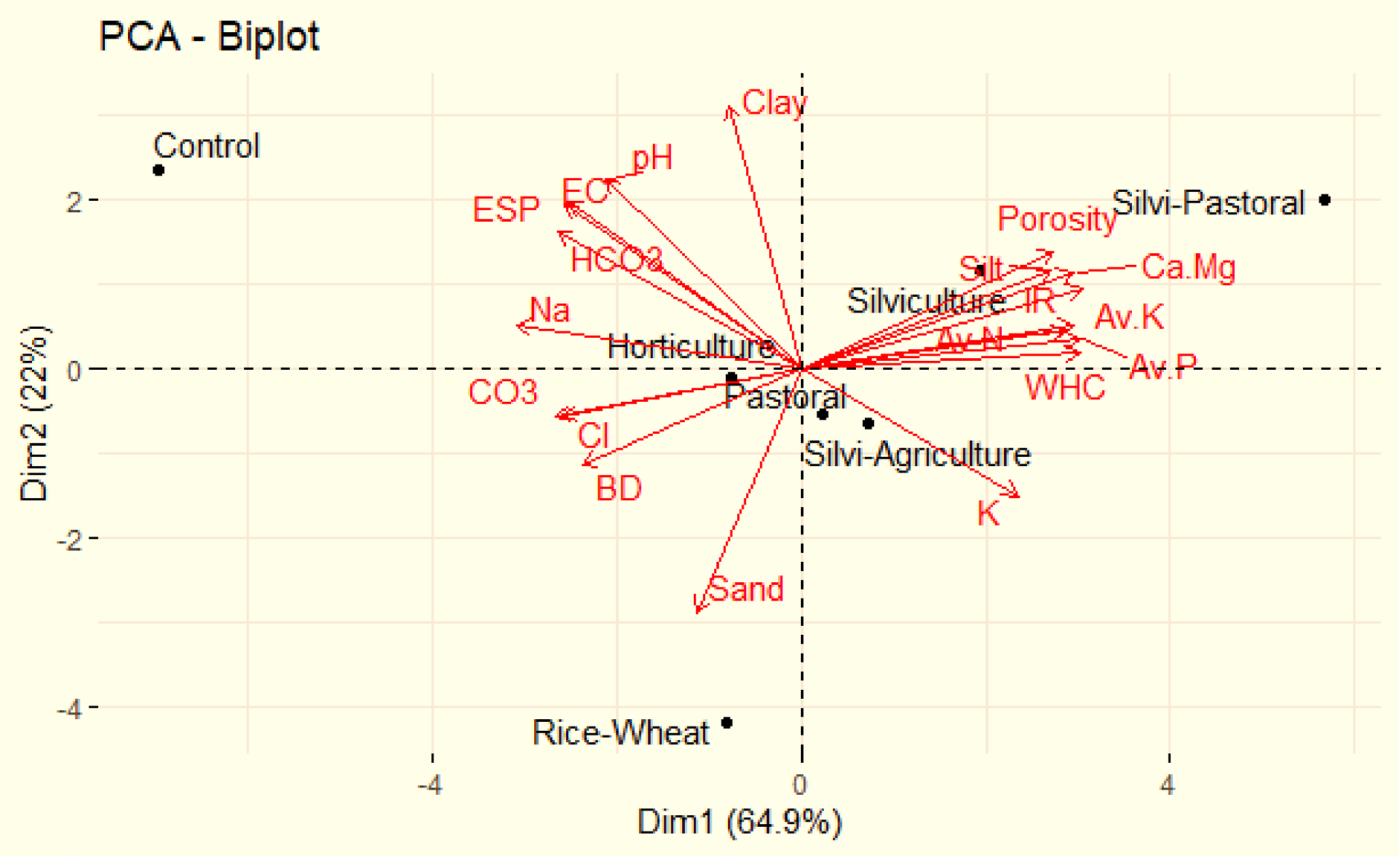
Principal component analysis (PCA) of soil properties under different land uses in salt affected soils.

The principal component analysis limits the variables and extracts smaller number of independent factors for deducing the association among observed variables (soil properties). Three principal components (PC1, PC2 and PC3) with eigenvalues greater than 1 were extracted (Table 8). The PCA led to reduction of the initial dimension of the dataset to three components, which explains 65.9% of the total dataset variance. The first component (PC 1) of the rotated matrix showed strong positive correlation with available nitrogen (> 0.90) while as good positive correlation (> 0.75, < 0.90) was observed with BD, WHC, IR, available K, Na and OC (Table 9). The first component showed moderate correlation (> 0.60, < 0.75) with Ca + Mg and CO_3_. The second component showed strong correlation with clay and pH and good correlation with K, HCO_3_, EC and ESP. Similarly, the third component (PC 3) showed moderate correlation with silt, porosity, available P, Ca + Mg and Cl, and good correlation with sand and available K (Fig. 7).

**Table 8:** Principal component analysis of soil parameters.

Parameters	PC1	PC2	PC3
Sand	− 0.315	− 0.596	− 0.719
Silt	0.453	− 0.114	0.842
Clay	0.046	0.952	0.278
BD	− 0.896	− 0.036	− 0.257
Porosity	0.524	− 0.045	0.819
WHC	0.837	− 0.344	0.363
IR	0.887	− 0.231	0.35
Av N	0.937	− 0.225	0.259
Av P	0.439	− 0.38	0.807
Av K	0.77	− 0.163	0.604
Ca + Mg	0.62	− 0.128	0.756
K	0.083	− 0.802	0.532
Na	− 0.751	0.547	− 0.344
Cl	− 0.274	0.276	− 0.842
CO_3_	− 0.654	0.215	− 0.484
HCO_3_	− 0.501	0.797	− 0.195
pH	− 0.085	0.947	− 0.287
EC	− 0.437	0.881	− 0.137
ESP	− 0.419	0.883	− 0.185
OC	0.863	− 0.163	0.448
Eigen values	13.188	4.244	1.465
Variance (%)	65.941	21.219	7.325
Cumulative variance (%)	65.941	87.161	94.485

*BD* bulk density, *WHC* water holding capacity, *IR* infiltration rate, *pH*_*2*_ *and EC*_*2*_ refers to soil: water suspension ratio of 1:2, *ESP* exchangeable sodium percentage, *OC* organic carbon.

**Table 9:** Pearson’s correlation matrix among soil properties.

Parameters	Sand	Silt	Clay	BD	Porosity	WHC	IR	Av *N*	Av *P*	Av K	Ca + Mg	K	Na	Cl	CO_3_	HCO_3_	pH	EC	ESP	OC
Sand	1																			
Silt	− 0.721	1																		
Clay	− 0.787*	0.139	1																	
BD	0.532	− 0.699	− 0.137	1																
Porosity	− 0.717	0.930**	0.196	− 0.718	1															
WHC	− 0.296	0.683	− 0.185	− 0.753	0.721	1														
IR	− 0.362	0.687	− 0.094	− 0.846*	0.786*	0.965**	1													
Av N	− 0.34	0.659	− 0.101	− 0.878**	0.704	0.970**	0.978**	1												
Av P	− 0.477	0.894**	− 0.114	− 0.548	0.901**	0.812*	0.772*	0.711	1											
Av K	− 0.575	0.855*	0.061	− 0.805*	0.886**	0.937**	0.928**	0.917**	0.896**	1										
Ca + Mg	− 0.678	0.936**	0.135	− 0.746	0.931**	0.833*	0.821*	0.802*	0.929**	0.969**	1									
K	0.027	0.628	− 0.597	− 0.231	0.49	0.51	0.396	0.388	0.751	0.518	0.588	1								
Na	0.174	− 0.714	0.387	0.783*	− 0.711	− 0.913**	− 0.898**	− 0.907**	− 0.802*	− 0.869*	− 0.806*	− 0.707	1							
Cl	0.466	− 0.787*	0.034	0.381	− 0.885**	− 0.668	− 0.674	− 0.547	− 0.933**	− 0.764*	− 0.801*	− 0.596	0.615	1						
CO_3_	0.368	− 0.645	0.048	0.53	− 0.686	− 0.913**	− 0.845*	− 0.822*	− 0.807*	− 0.858*	− 0.769*	− 0.407	0.702	0.748	1					
HCO_3_	− 0.172	− 0.461	0.655	0.471	− 0.464	− 0.749	− 0.685	− 0.69	− 0.682	− 0.653	− 0.595	− 0.804*	0.899**	0.504	0.522	1				
pH	− 0.323	− 0.413	0.828*	0.14	− 0.325	− 0.491	− 0.391	− 0.367	− 0.62	− 0.38	− 0.381	− 0.925**	0.682	0.514	0.389	0.828*	1			
EC	− 0.289	− 0.425	0.791*	0.424	− 0.405	− 0.701	− 0.648	− 0.64	− 0.63	− 0.549	− 0.475	− 0.808*	0.863*	0.489	0.509	0.939**	0.918**	1		
ESP	− 0.248	− 0.463	0.766*	0.396	− 0.391	− 0.722	− 0.623	− 0.64	− 0.664	− 0.58	− 0.521	− 0.863*	0.863*	0.481	0.553	0.945**	0.929**	0.980**	1	
OC	− 0.524	0.828*	0.012	− 0.934**	0.826*	0.912**	0.934**	0.954**	0.785*	0.954**	0.906**	0.478	− 0.910**	− 0.605	− 0.746	− 0.655	− 0.365	− 0.586	− 0.598	1

*B.D* bulk density, *WHC* water holding capacity, *IR* infiltration rate, *EC* electrical conductivity, *OC* soil organic carbon, *ESP* exchangeable sodium percentage, *Av. N* available nitrogen, *Av. P* available phosphorus, *Av. K* available potassium.

*Correlation is significant at the 0.05 level (2-tailed).

**Correlation is significant at the 0.01 level (2-tailed).

## Discussion

Present study revealed that the afforestation of sodium salt laden degraded sodic soils for more than 20 years showed a substantial reduction in salt content and improvement in physical, chemical, and biological properties, and favours in increasing SOC in surface and sub-surface layers and nutrient status of sodic soils compare to BGL and rice-wheat^42–44^. This is attributed to the enrichment of soil with the addition of organic matter, and thereby recycling of important nutrients^20,45^. In our study, soil physical properties like BD, porosity, and WHC influenced significantly with changing in LULCs. Among the different LULCs, highest improvement in soil physical properties was recorded under agroforestry based LULCs over the BGL and rice-wheat land uses. The highest improvement in these parameters after twenty years of study was recorded under *P. juliflora* based silvipastoral system. This may be attributed to the higher level of SOC in agroforestry LULCs. This result is in conformity with the results reported by workers^14,46–49^ in their earlier studies. The highest BD recorded in rice-wheat land use may be attributed to the exhaustive tillage operations in rice-wheat land use which may create a compact soil layer that increase BD^50^. Soil infiltration rate that is an important indicator of improvement in sodic soils has also influenced with LULCs. The improvement in this parameter ranges from 11.80 mm to 24.52 mm day^− 1^. This variation in infiltration rate may be attributed to differences in soil texture, BD and organic matter contents^51^. Lesser the organic matter, higher bulk density and declined porosity in rice-wheat and BGL results in poor soil structure and, thereby decreased infiltration rate^52^.

Soil chemical properties like pH, EC and ESP are the important parameters that decides the severity of the sodicity in the soil. In our study, the pH_1:2_ of surface soil (0–15 cm) in rice-wheat land use was significantly lower than that of land parcels covered under different agroforestry LULCs. This is due to reclamation of these parcels with chemical amendments like gypsum (CaSO_4_⋅2H_2_O) and continuous cultivation of rice-wheat crops^53,54^. However, highest reduction in this parameter in sub-surface (25–30 and 30–60 cm) soil layers recorded under silvipastoral land use^52,55^. This is because of replacement of exchangeable sodium by calcium due to the root exudates, and consequent leaching of sodium salts. Similarly, reduction in EC and ESP in surface soil (0–15 cm) was more pronounced in rice-wheat land use because of reclamation of this land parcel with gypsum (CaSO_4_⋅2H_2_O) and continuous cultivation of rice-wheat crops^56,57^. However, in sub-surface layers the reduction in these parameters were more in agroforestry LULCs than that of BGL and rice-wheat land uses. This may be due to high organic matter contents through decomposition of leaf litter, which mobilize the cations to neutralize the sodicity by reducing pH and evolution of CO_2_ that helps to mobilize the inherent Ca^2+^. This released Ca^2+^ can accelerate the reclamation by replacing the exchangeable Na^+^ from the soil^58^. This was more pronounced in silvipastoral land use, which proved more efficient in reducing soil pH, EC and ESP. *Prosopis* based land use significantly decreased exchangeable sodium percentage^59^. The organic carbon content is another important chemical characteristic of sodic soils. These soils are very poor (< 1.0 g kg^− 1^) in organic carbon content. The organic matter plays an important role in reduction of salt level^60,61^. After 20 years of study, it was found that the percent increase in organic carbon content in all the LULCs was higher in the surface (0–15 cm) layer and it declined with increasing depth. The highly significant increase in organic carbon was recorded in silvipastoral land use followed by silviculture and the lowest in BGL and rice-wheat land uses. The lower organic matter in rice-wheat and BGL compared to agroforestry land uses was most likely because of the less vegetation coverage. The trees continuously add organic matter through litter in the upper soil and increases root turnover^52,62,63^ which further increased SOC due to positive priming^64^. Our results are in conformity with the earlier findings reported^65,66^. Farooqi et al.^67^ links reclamation of salt-affected soils with increased carbon sequestration in soils. Lowest SOC in rice-wheat land use system in the present study may be due to removal of crop residues, intensive cultivation practices, and taking more microbial carbon from the soil system^68–70^. Declining in organic matter content may reduce soil quality as well as productivity of salt affected soils. Increasing area under rice-wheat land use may reduce the amount of organic matter and availability of essential nutrients in the soil resulting poor soil health and crop productivity. Organic matter content in soil plays an important role in the conversion and mineralization of nutrients in the soil. The highest mean available N content irrespective of soil depths was recorded under agroforestry LULCs, which was significantly higher over the BGL, and rice-wheat land uses (Table 6). This can be ascribed to the accumulation of organic residues in the form of leaf litter on the soil where BGL and rice-wheat land uses had lesser soil organic matter content than silvi-pastoral and silvicultural land uses. It has also been reported that the mineralization of the accrued soil organic matter to ammonia is the major sources of nitrogen in the soil^71^. Changes in land uses resulted in reduction of total nitrogen may affect the fertility and productivity of sodic soils because nitrogen is an essential nutrient for plant growth. In silvipastoral systems, trees help in maintaining soil fertility, cycle nutrients, improve microclimate and improve overall system productivity^72^. Improved soil fertility in agroforestry LULC having tree canopies may result from litter fall or dung inputs from sheltering animals^59^. Similarly, the increment in available P and K contents were recorded under agroforestry LULCs. This may be due to decreasing in soil pH and ESP and increasing soil organic matter contents, which may increase the availability of P and K to the plants. Additionally, the organic acid production during breakdown of organic materials increases P release. Soil quality improvement under *P. juliflora* in degraded lands including sodic lands has been reported from Indo-Gangetic plain region^73^.

Soil microbial properties like MBC, MBN and MBP, which are an indicator of microbial activities in the soil changes due to different LULCs. The highest improvement in these parameters was recorded under agroforestry LULCs. This may be attributed to the more organic matter in the top humus soil that promotes microbial activities^57,74^. MBC is a complex sign of changes in SOC because it has a much faster rate of turnover. Previous land use studies also reported significant reduction in MBC using conventional practices for rice-wheat land use as compared to forestland use^75–78^. Dehydrogenase activities (DHA) of the soil described the oxidative activity of soil microorganisms also influenced with LULCs. The higher DHA values recorded under silvipastoral and pastoral LULCs may be attributed to the assortment in vegetation, improvement in soil properties and environmental factors affecting soil properties. However, low DHA values in BGL and rice–wheat land uses were due to low SOC and repressing effects of mineral fertilizers as observed in present study. The organic matter added through leaf litter could also be a contributing factor to motivate DHA by increasing availability of substrate for the soil microbial community in agroforestry land uses^79,80^.

Among the ionic contents analyzed in this study, Na^+^ content, which was highly dominant in surface soils, decreased in all the LULCs. After twenty years of different LULCs, it decreased in surface layers (0–15 cm) and accumulated in the lower levels due to leaching of salts and dissolution of CaCO_3_ by the plant roots in the presence of CO_2_, causing replacement of adsorbed Na^+^. The deep-rooted trees in silvipasture and silviculture land uses could improve the nutrient status of degraded soil by redistributing nutrients from the deeper soil layers.

## Conclusions

This study evaluated the impact of different land use–land cover (LULC) systems on the physico-chemical and biological properties of degraded sodic soils. Results showed that long-term agroforestry practices significantly improved soil health by increasing SOC, available N, P, K, microbial biomass (C, N, P), and dehydrogenase activity, while reducing soil pH and ESP. Among all systems, the silvipastoral system—*P. juliflora* with salt-tolerant grasses (*L. fusca*, *T. alexandrinum*)—was most effective in enhancing soil fertility and microbial activity. Conversely, traditional rice–wheat systems exhibited poor soil health due to low organic inputs and intensive cultivation. Overall, the findings highlight agroforestry, particularly silvipastoral land use, as a nature-based solution for restoring and sustaining sodic soils. Although, the present study is confined to represent sodic soils dominant in the Indo-Gangetic plains but may be useful for the other geographical areas having similar degraded lands. The study will be useful in strategizing nature based solution for rejuvenation of sodic lands through agroforestry land uses and management of soil fertility and ecological sustainability.

## Data Availability

The datasets used and/or generated during the current study are available from the corresponding author on reasonable request.

## References

[1] Singh, P., Kumar, V. & Mathur, A. Comparative study of sodic wastelands and water-logged area using IRS P6 LISS- III and LISS IV data through the GIS techniques. *Int. J. Eng. Res. Tech.* **2** (9), 1628–1639. 10.17577/IJERTV2IS90588 (2013).

[2] FAO. *Global Status of Salt-Affected Soils—Main Report*. 10.4060/cd3044en (2024).

[3] Kumar, R. et al. Reclamation of salt-affected soils in india: Progress, emerging challenges, and future strategies. *Land. Degrad. Dev.* **33** (13), 2169–2180. 10.1002/ldr.43202180 (2022).

[4] Shukla, S. K., Singh, K., Singh, B. & Gautam, N. N. Biomass productivity and nutrient availability of *Cynodon dactylon* (L.) Pers. Growing on soils of different sodicity stress. *Bio Bioeng.* **35**, 3440–3447 (2011).

[5] Singh, K., Pandey, V. C., Singh, B. & Singh, R. R. Ecological restoration of degraded sodic lands through afforestation and cropping. *Ecol. Eng.* **43**, 70–80 (2012).

[6] Hanay, A., Buyuksonmez, F., Kiziloglu, F. M. & Canbolat, M. Y. Reclamation of saline sodic soils with gypsum and MSW compost. *Compost Sci. Util.* **12**, 175–179 (2004).

[7] Bhardwaj, A. K., Mandal, U. K., Bar-Tal, A., Gilboa, A. & Levy, G. J. Replacing saline sodic irrigation water with treated wastewater: effects on saturated hydraulic conductivity, slaking, and swelling. *Irrig. Sci.* **26**, 139–146 (2008).

[8] Laudicina, V. A., Novara, A., Barbera, V., Egli, M. & Badalucco, L. Long-term tillage and cropping system effects on chemical and biochemical characteristics of soil organic matter in a mediterranean semi-arid environment. *Land. Degrad. Dev.* **26**, 45–53. 10.1002/ldr.2293 (2015).

[9] Wildemeersch, J. C. J., Garba, M., Sabiou, M., Sleutel, S. & Cornelis, W. The effect of water and soil conservation (WSC) on the soil chemical, biological, and physical quality of a plinthosol in Niger. *Land. Degrad. Dev.* **26**, 773–783. 10.1002/ldr.2416 (2015).

[10] Aksakal, E. L., Sari, S. & Angin, I. Effects of vermicomposting application on soil aggregation and certain physical properties. *Land. Degrad. Dev.* **27**, 983–995 (2016).

[11] Zhang, A. et al. Nursery-box total fertilization technology (NBTF) application for increasing nitrogen use efficiency in Chinese irrigated rice land: N-soil interactions. *Land. Degrad. Dev.* **27**, 1255–1265. 10.1002/ldr.2346 (2016).

[12] Muñoz-Rojas, M., Jordán, A., Zavala, L. M., De la Rosa, D. & Abd-Elmabod, S. K. Anaya Romero, M. Impact of land use and land cover changes on organic carbon stocks in mediterranean soils (1956–2007). *Land. Degrad. Dev.* **26** (2), 168–179. 10.1002/ldr.2194 (2015).

[13] Mandal, A. K., Sharma, R. C. & Singh, G. Assessment of salt affected soils in India using GIS. *Geo Int.* **24** (6), 437–456. 10.1080/10106040902781002 (2009).

[14] Dutta, A., Basak, N., Chinchmalatpure, A. R., Banyal, R. & Chaudhari, S. K. Land-use influences soil properties of sodic land in Northwest India. *J. Soil. Salinity Water Qual.* **9** (2), 178–186 (2017).

[15] Mandal, A. K., Arora, S., Sharma, P. C. & Yadav, R. K. Spatial assessment, mapping, and characterization of salt–affected soils in Uttar Pradesh state of the gangetic plain (IGP), India, for planning reclamation and management. *Environ. Monit. Assess.* **197**, 739 (2025). 40481355 10.1007/s10661-025-14158-4

[16] Sharma, D. K. et al. Assessment of production and monetary losses from salt-affected soils in India. *Technical Bulletin: ICAR-CSSRI/Karnal/2015/05* (ICAR-Central Soil Salinity Research Institute, 2015).

[17] Arora, S. & Singh, B. P. Status of soil degradation in state of Uttar Pradesh. *J. Soil. Water Conserv. India*. **19** (2), 119–125 (2024).

[18] Thimmappa, K., Tripathi, R. S. & Singh, Y. P. Livelihood security of resource poor farmers through alkali land reclamation: an impact analysis. *Agri Eco Res. Rev.* **26**, 139–147 (2013).

[19] Qadir, M., Oster, J. D., Schubert, S., Noble, A. D. & Sahrawat, K. L. Phytoremediation of sodic and saline-sodic soils. *Adv. Agron.* **96**, 197–247 (2007).

[20] Singh, Y. P., Singh, G. & Sharma, D. K. Ameliorative effect of multipurpose tree species grown on sodic soils of Indo-Gangetic alluvial plains of India. *Arid Land. Res. Manag*. **25**, 55–74. 10.1080/15324982.2010.528150 (2011).

[21] De Blécourt, M., Brumme, R., Xu, J., Corre, M. D. & Veldkamp, E. Soil carbon stocks decrease following conversion of secondary forests to rubber (*Hevea Brasiliensis*) plantations. *PLoS ONE*. **8**, e69357. 10.1371/journal.pone.0069357 (2013). 23894456 10.1371/journal.pone.0069357 PMC3716606

[22] Ahrends, A. et al. Current trends of rubber plantation expansion May threaten biodiversity and livelihoods. *Global Environ. Change*. **34**, 48–58 (2015).

[23] Guillaume, T., Damris, M. & Kuzyakov, Y. Losses of soil carbon by converting tropical forest to plantations: erosion and decomposition estimated by δ C. *Global Changes Bio*. **21**, 3548–3560. 10.1111/gcb.12907 (2015). 10.1111/gcb.12907 25707391

[24] Nath, A. J., Brahma, B., Sileshi, G. W. & Das, A. K. Impact of land use changes on the storage of soil organic carbon in active and recalcitrant pools in a humid tropical region of India. *Sci. Total Env*. **624**, 908–917. 10.1016/j.scitotenv.2017.12.199 (2018). 29275253 10.1016/j.scitotenv.2017.12.199

[25] González, A. P., de Abreu, C. A. & Tarquis, A. M. Medina Roldan, E. Impacts of land use changes on soil properties and processes. *Sci. World J.* **1**, 831975. 10.1155/2014/831975 (2014). 10.1155/2014/831975 PMC4294292 25614897

[26] Sharma, R. C., Singh, R., Singh, Y. P. & Singh, G. *Sodic Soil of Shivri Experimental Farm; Site Characteristics, Reclamability and Use Potential Different Land Uses* Vol. 36 (Central Soil Salinity Research Institute, 2006).

[27] Blake, G. R. & Hartge, K. H. Bulk density. In *Methods of Soil Analysis. Part 1—Physical and Mineralogical Methods* (ed Klute, A.), 2 edn., 363–382 (American Soc. Agro. Soil Sci. Soc. America, 1986).

[28] Piper, C. S. *Soil and Plant Analysis* (Hans’s, 1966).

[29] Brechtel, H. M. *Application of an Inexpensive Double Ring Infiltration: Hydrological Techniques for Upstream Conservation. Conservation Guide* (FAO, 1976).

[30] Yadav, Y. P. & Vasistha, H. B. Infiltration capacity of forest soils under *Cryptomeria Japonica*. *Indian Forester*. **115**, 435–441 (1989).

[31] Jackson, M. L. *Soil Chemical Analysis* 183–226 (Prentice Hall of India, 1967).

[32] Richards, L. A. *Diagnosis and Improvement of Saline and Sodic Soils. USSL Handbook 60* (1954).

[33] Walkley, A. & Black, T. A. An examination of the Degtjareff method for determining soil organic matter and a proposed modification of the chromic acid Titration method. *Soil. Sci.* **37**, 29–38 (1934).

[34] Subbiah, B. V. & Asija, G. L. A rapid procedure for Estimation of available nitrogen in soils. *Curr. Sci.* **25**, 259–263 (1956).

[35] Olsen, S. R. & Dean, L. A. Phosphorus. In *Methods of Soil Analysis* (ed Black, C. A.) 1035–1049 (1965).

[36] Cheng, K. L. & Bray, R. H. Determination of calcium and magnesium in soil and plant material. *Soil Sci* **72**, 449–458 (1951).

[37] Vance, E. D., Brookes, P. C. & Jenkinson, D. S. An extraction method for measuring soil microbial biomass C. *Soil. Bio Biochem.* **19**, 703–707 (1987).

[38] Brookes, P. C., Landman, A., Pruden, G. & Jenkinson, D. S. Chloroform fumigation and the release of soil nitrogen: a rapid direct extraction method to measure microbial biomass nitrogen in soil. *Soil. Bio Biochem.* **17**, 837–842 (1985).

[39] Brookes, P. C., Powelson, D. S. & Jenkinson, D. S. Measurement of microbial biomass phosphorus in soil. *Soil. Bio Biochem.* **14**, 319–329 (1982).

[40] Klein, D. A., Loh, T. C. & Goulding, R. L. A rapid procedure to evaluate dehydrogenase activity of soils low in organic matter. *Soil. Bio Biochem.* **3**, 385–387 (1971).

[41] Kaiser, H. F. The application of electronic computers to factor analysis. *Edu Psycho Measure*. **20**, 141–151 (1960).

[42] Wong, V. N. L., Murphy, B. W., Koen, T. B., Greene, R. S. B. & Dalal, R. C. Soil organic carbon stocks in saline and sodic landscapes. *Aust J. Soil. Res.* **46**, 378–389 (2008).

[43] Sharma, R. C., Rao, B. R. M. & Saxena, R. K. Salt affected soils in India-current assessment. In *Advances in Sodic Land Reclamation. International Conference on Sustainable Management of Sodic Lands, Held on 9–14 February at Lucknow, India* 1–26 (2004).

[44] Dalal, R. C., Wong, V. N. L. & Sahrawat, K. L. Salinity and sodicity affect organic carbon dynamics in soil. *Bull. Indian Soc. Soil. Sci.* **28**, 95–117 (2011).

[45] Singh, Y. P. et al. Harnessing productivity potential and rehabilitation of degraded sodic lands through Jatropha based intercropping systems. *Agri Eco Env*. **233**, 121–129. 10.1016/j.agee.2016.08.034 (2016).

[46] Singh, Y. P. et al. Evaluation of *Jatropha Curcas* genotypes for rehabilitation of degraded sodic lands. *Land. Deg Dev.* **26**, 510–520. 10.1002/ldr.2398 (2015).

[47] Singh, Y. P. et al. Jatropha based intercropping system: an alternate land use for rehabilitation of degraded sodic lands. *Indian J. Agri Sci.* **9** (1), 9–15 (2019).

[48] Singh, Y. P., Arora, S., Mishra, V. K. & Bharadwaj, A. K. Regaining the agricultural potential of sodic soils and improved smallholder food security through integration of gypsum, Pressmud and salt tolerant varieties. *Agro Sust Food Syst.* **46**, 410–431. 10.1080/21683565.2021.2015735 (2022).

[49] Kisku, T. K. et al. Evaluation of saturated hydraulic conductivity from soil properties in an inceptisol using different land cover and depths. *J. Appl. Nat. Sci.* **9** (3), 1482–1488. 10.31018/jans.v9i3.1388 (2017).

[50] Meena, V. S. et al. Land use changes: strategies to improve soil carbon and nitrogen storage pattern in the mid-Himalaya ecosystem, India. *Geoderma* **321**, 69–78 (2018).

[51] Singh, Y. P., Arora, S., Mishra, V. K., Dixit, H. & Gupta, R. K. Conjoint use of chemical amendments and municipal solid waste compost for amelioration of degraded sodic soil. *J. Indian Soc. Soil. Sci.* **66** (4), 392–398 (2018).

[52] Singh, Y. P., Singh, G. & Sharma, D. K. Bio-amelioration of alkali soils through agroforestry systems in central Indo-Gangetic plains of India. *J. Forestry Res.* **25** (4), 887–896. 10.1007/s11676-014-0535-1 (2014).

[53] Basak, N. et al. Distribution and characteristics of salt-affected soils of Mahendragarh district in Haryana. *J. Soil. Salinity Water Qual.* **8** (2), 153–160 (2016).

[54] Singh, Y. P. et al. Restoration of ecosystem services through afforestation on degraded sodic lands in Indo-Gangetic plains. *Indian J. Agric. Sci.* **89** (9), 1492–1497 (2019).

[55] Singh, Y. P., Mishra, V. K., Arora, S., Dagar, J. C. & Lal, K. Restoration of degraded sodic soils through silvipastoral systems in Indo-Gangetic plains. *Land. Deg Dev.* **33** (9), 1459–1473. 10.1002/ldr.4222 (2022).

[56] Singh, Y. P., Singh, G. & Sharma, D. K. Biomass and bio-energy production of ten multipurpose tree species planted in sodic soils of Indo-Gangetic plains. *J. Forestry Res.* **21** (1), 19–24. 10.1007/s11676-010-0003-5 (2010).

[57] Singh, Y. P., Singh, G. & Sharma, D. K. Performance of pastoral: silvipastoral and silvicultural systems in alkali soils of Indo-Gangetic plains. *J Soil. Water Conserv India*. **14**, 168–173 (2015).

[58] Singh, Y. P., Arora, S., Mishra, V. K., Dixit, H. & Gupta, R. K. Composting of municipal solid waste and farm wastes for its use as amendment in sodic soil. *J. Soil. Water Conserv. India*. **16** (2), 172–177 (2017).

[59] Shiferaw, W., Demissew, S., Bekele, T., Aynekulu, E. & Pitroff, W. Invasion of *Prosopis Juliflora* and its effects on soil physicochemical properties in Afar region, Northeast Ethiopia. *Int. Soil. Water Conserv. Res.* **9** (4), 631–638 (2021).

[60] Ritchie, G. S. P. & Dolling, P. J. The role of organic matter in soil acidification. Australian *J. Soil Res.* **23**, 569–576. 10.1071/SR9850569 (1985).

[61] Singh, Y. P. et al. Evaluation of multipurpose tree species for managing degraded sodic land ecosystem in Indo-Gangetic alluvial plains. *Trop. Eco*. **59** (4), 701–714 (2018).

[62] Kimmins, J. P. *Forest Ecology: A Foundation for Sustainable Forest Management and Environment Ethics in Forestry*, 3rd edn, 611 (Prentice Hall, 2004).

[63] Jha, P. et al. Soil carbon pools, mineralization and fluxes associated with land use change in vertisols of central India. *Natl. Aca Sci. Lett.* **35**, 475–483 (2012).

[64] Wu, J. T., Wu, S. C., Hajj, G. A., Bertiger, W. I. & Lichten, S. M. Effects of antenna orientation on GPS carrier phase. *Manu Geo*. **18**, 91–98 (1993).

[65] Soleimani, A., Hosseini, S. M., Bavani, A. R. M., Jafari, M. & Francaviglia, R. Influence of land use and land cover change on soil organic carbon and microbial activity in the forest of Northern Iran. *Catena* **177**, 227–237. 10.1016/j.catena.2019.02.018 (2019).

[66] Moges, A., Dagnachew, M. & Yimer, F. Land use effects on soil quality indicators: A case study of Abo-Wonsho Southern Ethiopia. *Appl. Environ. Soil. Sci.* **1**, 784989. 10.1155/2013/784989 (2013).

[67] Farooqi, Z. U. R., Sabir, M., Ahmad, H. R., Shahbaz, M. & Smith, J. Reclaimed salt-affected soils can effectively contribute to carbon sequestration and food grain production: evidence from Pakistan. *Appl. Sci.* **13** (3), 1436. 10.3390/app13031436 (2023).

[68] Huang, J. & Song, C. Effects of land use on soil water soluble organic C and microbial biomass C concentrations in the Sanjiang plain in Northeast China. *Acta Agri Scand. Sect. B-Soil Plant. Sci.* **60** (2), 182–188. 10.1080/09064710802680387 (2010).

[69] Sharma, V., Hussain, S., Sharma, K. R. & Arya, V. M. Labile carbon pools and soil organic carbon stocks in the foothill Himalayas under different land use systems. *Geoderma* **232–234**, 81–87. 10.1016/j.geoderma.2014.04.039 (2014).

[70] Reza, S. K., Baruah, U., Nayak, D. C., Dutta, D. & Singh, S. K. Effects of land-use on soil physical, chemical and microbial properties in humid subtropical North Eastern India. *Natl. Acad. Sci. Lett.* **41** (3), 141–145. 10.1007/s40009-018-0634-1 (2018).

[71] Galloway, J. N., Dentener, F. J. & Capone, D. G. Nitrogen cycles: past, present, and future. *Biogeo* **70**, 153–226. 10.1007/s10533-004-0370-0 (2004).

[72] Tiessen, H., Menezes, R. S. C., Salcedo, I. H. & Wick, B. Organic matter transformations and soil fertility in a tree pasture in semiarid NE Brazil. *Pl Soil.* **252**, 195–205 (2003).

[73] Edrisi, S. A., El-Keblawy, A. & Abhilash, P. C. Sustainability analysis of prosopis Juliflora (Sw.) DC based restoration of degraded land in North India. *Land* **9** (2), 59. 10.3390/land9020059 (2020).

[74] Kumar, U. et al. Variation of functional diversity of soil microbial community in subhumid tropical rice-rice cropping system under long-term organic and inorganic fertilization. *Ecolo Indica*. **73**, 536–543. 10.1016/j.ecolind.2016.10.014 (2017).

[75] Fierer, N., Schimel, J. P. & Holden, P. A. Variations in microbial community composition through two soil depth profiles. *Soil. Bio Biochem.* **35**, 167–176. 10.1016/S0038-0717(02)00251-1 (2003).

[76] Fall, D. et al. Effect of distance and depth on microbial biomass and mineral nitrogen content under *Acacia Senegal* (L.) Wild. Trees. *J. Env Manag*. **95**, S260–S264. 10.1016/j.jenvman.2011.03.038 (2012). 10.1016/j.jenvman.2011.03.038 21514716

[77] Reza, S. K., Baruah, U., Nath, D. J., Sarkar, D. & Gogoi, D. Microbial biomass and enzyme activity in relation to shifting cultivation and horticultural practices in humid subtropical North-Eastern India. *Range Manag Agrofor.* **35**, 78–84 (2014).

[78] Zuber, S. M. & Villamil, M. B. Meta-analysis approach to assess effect of tillage on microbial biomass and enzyme activities. *Soil. Bio Biochem.* **97**, 176–187. 10.1016/j.soilbio.2016.03.011 (2016).

[79] Kuwano, B. H., Knob, A. & Fagotti, D. S. L. Soil quality indicators in a Rhodickandiudult under different land uses in Northern Parana, Brazil. *Rev. Bras. Ci Solo*. **38** (1), 50–59. 10.1590/S0100-06832014000100005 (2014).

[80] Blonska, E., Lasota, J. & Zwydak, M. The relationship between soil properties, enzyme activity and land use. *For. Res.* **78**, 39–44 (2017).

